# Tumour-reactive plasma cells in antitumour immunity: current insights and future prospects

**DOI:** 10.1093/immadv/ltae003

**Published:** 2024-04-25

**Authors:** Peng Chen, Yiwei Chu, Ronghua Liu

**Affiliations:** Shanghai Fifth People’s Hospital and Key Laboratory of Medical Epigenetics and Metabolism, Institutes of Biomedical Sciences, Fudan University, Shanghai, China; Department of Immunology, School of Basic Medical Sciences, and MOE Innovative Center for New Drug Development of Immune Inflammatory Diseases, Fudan University, Shanghai, China; Shanghai Fifth People’s Hospital and Key Laboratory of Medical Epigenetics and Metabolism, Institutes of Biomedical Sciences, Fudan University, Shanghai, China

**Keywords:** plasma cell, tumour-infiltrating B cell, tumour-specific antigen, antibody

## Abstract

Tumour-reactive plasma cells (TRPCs) have been reported to be positively associated with the long-term survival of patients with various cancers. However, unlike tumour-specific antigen (TSA)-induced T cells which have precise effects against tumours, plasma cells require TSA to obtain specific responses. Therefore, the search for a TSA suitable for B-cell recognition is urgent. In this review, we discuss the functions of tumour-reactive plasma cells. Further, this review also explores the concept of screening for neoantigen-reactive plasma cells, drawing inspiration from T-cell screening methods. While challenges exist, such as epitope prediction and efficient screening, the development of novel techniques may lead to the discovery of highly specific plasma cells for adoptive cell therapy. In conclusion, tumour-reactive plasma cells are emerging as powerful players in cancer immunotherapy. Their ability to produce antibodies against a variety of antigens, especially neoantigens, opens new avenues for personalised treatments. Overcoming challenges in epitope prediction and screening will be crucial in harnessing the full potential of these plasma cells for the benefit of cancer patients.

## Introduction

B cell, acting as antigen presentation cell and antibody secreting cell, is well known in the field of immunology. Function of B cell in tumour has been controversial for many years, but its antitumour effect has been reported frequently in many cancers [1]. Tumour-infiltrating B cells, including B cells and plasma cells, could form tumour-associated immune aggregates from small unorganized clusters to tertiary lymphoid structures (TLSs) [2, 3]. TLSs are found to generate tumour-specific antibodies and help improve survival in some cancers [4, 5]. However, plasma cells, an important role in humoral immunity, seem to be underestimated in anti-tumour response, which promote the formation of TLSs, secret IgG1, drive cytotoxic T-cell responses [3]. Some tumour-reactive plasma cells and antibodies have been found in head and neck squamous cell carcinoma (HNSCC) and ovarian carcinoma to target on tumour cells [6, 7]. With the guidance of second-generation high-throughput sequencing technology, more and more neoantigens are found with high immunogenicity [8]. The preparation of tumour-specific plasma cells and antibodies has become a trend in response to the growing demand for personalised treatment. Consequently, there is a rising trend towards the preparation of tumour-specific plasma cells and antibodies, driven by the growing demand for personalised treatments. These recent developments highlight the substantial potential of plasma cells in antitumour immunity. While some excellent reviews have been recently published on tumour infiltrating B cells, in this review, we bring the focus on the functions of tumour-reactive plasma cells. Specifically, we review the latest research in the field specifically pointing to the latest research on adding to the function tumour-reactive plasma cells, latest improvements made on antigens to better induce plasma cells and finally propose a strategy for screening tumour-specific antigen (TSA)-reactive plasma cells.

## Function of tumour-reactive plasma cell

### Tumour-reactive plasma cell promotes tumour elimination

As the tumour progresses and spreads, tumour cells can infiltrate the surrounding tissues and organs and eventually enter the lymphatic system. The presence of tumour-associated B cells in various cancer types, including breast, liver, colon, and others, has shown a positive correlation with improved prognosis [9–11]. B cells can act as antigen-presenting cells to activate CD4+ T cells while also secreting a series of cytokines (including TNF, IL-2, IL-6, and IFN-γ) to activate and recruit other immune effector cells [12]. Within the tumour microenvironment, a fraction of infiltrating lymphocytes comprises tumour-reactive plasma cells. These plasma cells are unique due to their location within the tumour and their active involvement in the tumour immune response. They also produce a large amount of proinflammatory cytokines, including TNF-α, IL-17, GM-CSF, and antibodies, which can induce, activate and interact with other immune cells [13]. B cells that differentiate into plasma cells after exposure to antigens generate tumour-reactive antibodies (TRAs). These antibodies, along with processes like antibody-dependent cellular cytotoxicity (ADCC), complement activation, neutralisation, and high levels of B-cell and plasma cell characteristic genes, have been linked to increased overall survival in patients with melanoma, lung adenocarcinoma, pancreatic adenocarcinoma, and HNSCC [3].

Previous studies have reported that the prognostic value of tumour-infiltrating lymphocytes (TILs), specifically of CD3+ and/or CD8+ is higher when tumour-infiltrating B lymphocytes (TIL-Bs) and/or plasma cells (PC) are present. These results are consistent with studies evaluating the prognostic value of tertiary lymphoid structures (TLS) and are indicative of a strong TIL reaction [14]. Wouters *et al*. review summarising 69 studies across 19 human cancer types provided useful insights into the prognostic significance of TIL-B and/or PC. This review found that most studies reported a positive or neutral prognostic impact, with only a few reporting negative effects [1]. Thus, TIL-Bs appear to hold considerable significance for cancer prognosis.

Research has shown that both TLSs and lymphoid aggregates (LAs) are surrounded by plasma cells. For example, a study by Patil *et al*. using multiple immunofluorescence (mIF) techniques reported a close relationship between plasma cells and CD3+ T-cell infiltration, tumour-associated macrophages (CD68+ or CD163+), and overall B cell (CD20+) infiltration [15]. These observations suggest that plasma cell infiltration may be associated with T-cell infiltration in TLS or LA and may contribute to the formation of these structures. However, the specific mechanism remains poorly understood.

### Involvement of antibodies in the plasma cell antitumour reaction

Tumour-reactive antibodies (TRAs), which exist in both patient serum and the tumour microenvironment, have the capacity to recognise a wide range of antigens. These antigens included overexpressed or aberrantly expressed self-antigens, abnormally modified proteins, and normal molecules [3]. An early study has reported that antibodies against melanoma-associated antigen (MAGE) and tyrosinase can be detected in the serum of patients with melanoma [16]. This study also reported that antibodies against TAA HER2/neu can be detected in breast cancer patients, and antibodies against the testis-cancer antigen NY-ESO-1 can be detected in ovarian cancer patients. Recent studies have identified the presence of TRAs. For example, in cases of HPV-induced head and neck squamous cell carcinoma, a group of antibody-secreting cells (ASCs) were found to exist in the tumour microenvironment and produce strong antitumour reactions against HPV E2 protein-specific IgG antibodies [6].

The use of tumour-related antibodies as serum biomarkers for early clinical diagnosis is gaining popularity [17]. Many tumour antigen antibodies have been used in cancer treatment. For example, the monoclonal antibody drug herceptin, which targets HER2/neu, has ushered in an era of targeted therapy for breast cancer and a wave of monoclonal antibody therapy for solid tumours. The emergence of anti-PD-1 and anti-PD-L1 drugs has led to significant advancements in treating cancer. However, more than half of the patients do not improve after treatment with anti-PD-1/PD-L1 antibodies, due to T-cell inactivation limitation or imbalanced tumour burden [18].

Tumour-reactive antibodies (TRAs), primarily IgG1, are secreted by tumour-reactive plasma [3]. Studies have shown that B cells within tumours can differentiate into ASCs, including both autoreactive and tumour-specific types. Tumour-specific ASCs are predominantly derived from somatic hypermutation (SHM)and exhibit a high degree of diversity driven by specific antigens [7]. Further research has shown that ASCs within the tumour microenvironment have the capability to produce antibodies that target tumours. Specific antibodies against matrix metalloproteinases 14 (MMP14) have been identified in advanced ovarian serous tumours, providing a solid theoretical basis for the development of new antigen-reactive plasma cells and antibodies.

Currently, almost no antibody drugs targeting new antigens are available, with the majority still in the preclinical research stage. For example, in 2018, Frontier Biotech developed GT90001 (ascrinvacumab), a human monoclonal antibody targeting activin receptor-like kinase-1 (ALK-1). In October 2021, this drug received approval for clinical use in combination with another medication, OPDIVO (nivolumab), for patients with advanced liver cancer who had not previously undergone systemic therapy. This combination therapy has shown an objective response rate (ORR) of up to 40%. Therefore, the development of antigen-reactive antibodies based on new antigens holds significant research potential and promising application prospects.

## Antigens for inducing tumour reactive plasma cells

### Insufficiency of plasma cells in clinical treatment

Patients with triple-negative breast cancer who have above-median densities of CD38+ plasma cells have significantly better disease-free survival [19]. However, some studies have shown that plasma cells are associated with poor prognoses [20, 21]. This suggests bidirectional regulation of tumour plasma cells.

Chronic inflammation dependent on B cells promotes cancer progression, and involves pro-inflammatory factor induction by antigen–antibody complexes. Further, the complementary pathway promoting angiogenic factors plays an important role [22–24]. Circulating immune complexes (CICs) in patients with genitourinary cancers are associated with poor prognosis [25]. These CICs may have the ability to bind to bone marrow cells within the tumour, activating Fcγ receptors on these cells, thereby inducing the activity of myeloid suppressor cells that promote tumour development [26].

Research has shown that mutations and accumulation of the p53 protein increase its immunogenicity, making anti-p53 antibodies a potential clinical indicator [27]. However, the regression of lung tumours during treatment is correlated with low levels of anti-p53 antibodies, suggesting that this may be due to immunogenic deprivation [28]. Further, during chemotherapy, tumour cell lysis leads to increased exposure to p53 antigens, which may increase the production of anti-p53 antibodies [29]. A high titre of anti-p53 antibodies (titre ratio >5) is associated with a survival advantage, and requires continuous stimulation of the immune system for maintenance [30, 31].

These findings are closely related to the function of plasma cells. Therefore, improving the anti-tumour function of plasma cells may improve their ability to attack tumours and reverse their tumour-promoting effects.

### Optimised antigen improves the antitumour effect of plasma cell

**Figure 1.**
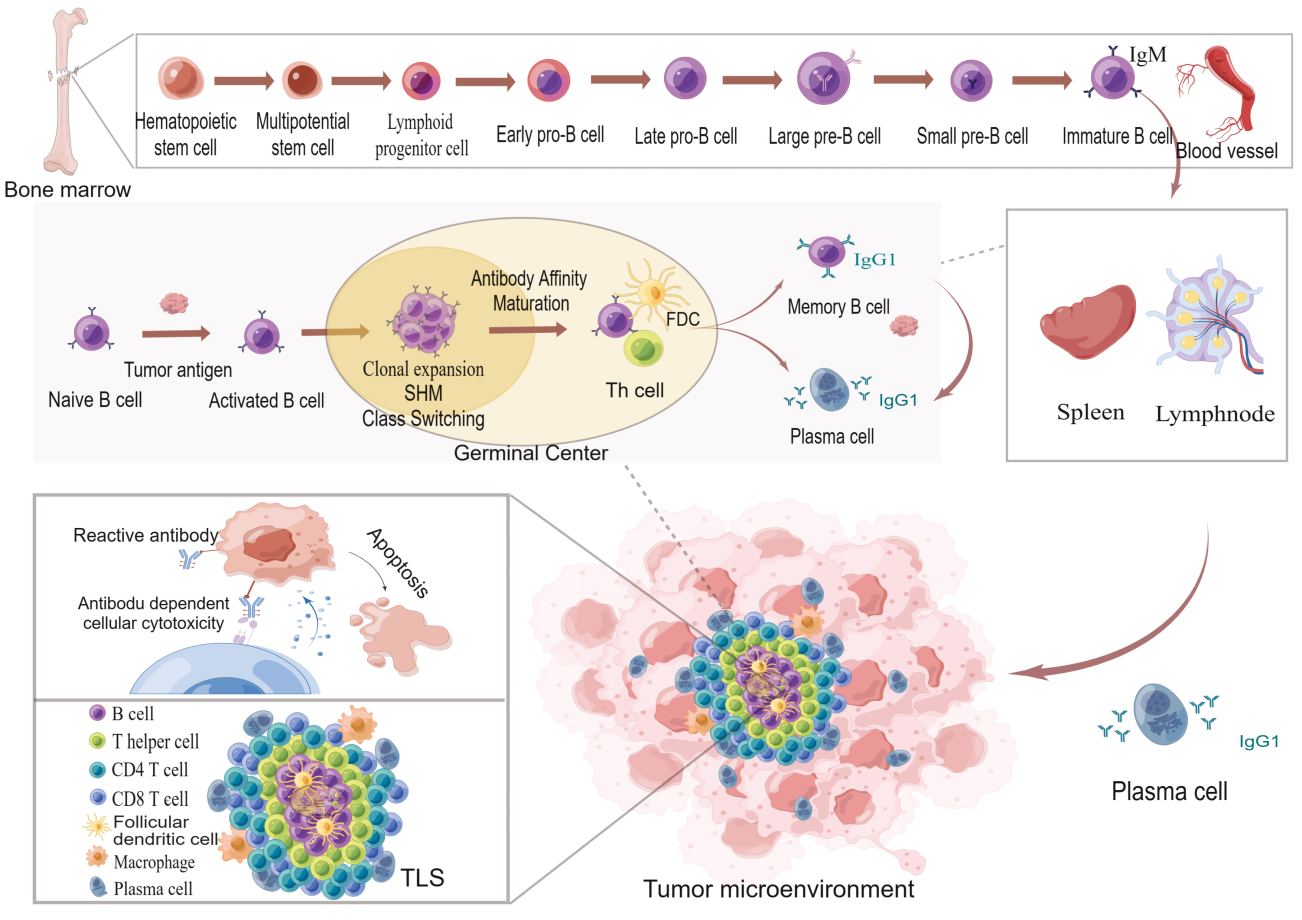
Development and differentiation of tumour-reactive plasma cell. Haematopoietic stem cells arise from bone marrow and undergo continuous differentiation to pro-B cells and eventually to pre-B cells, which then produce IgM and express it on their cell surface, becoming immature B cells. These B cells later migrate via blood vessels to secondary lymphoid organs. Naive B cells within the spleen and lymph nodes are activated following stimulation by free tumour antigens and proceed to clone and expand in the germinal centre, undergoing somatic hypermutation (SHM) and class switching. Only B cells with high antigen affinity are able to interact with more antigens on the surface of follicular dendritic cells, take up the antigen, and present it through MHC-II pathway. Then helper T cells recognise them and activate B cells. This process is repeated for about 2–3 weeks until B cells producing high-affinity antibodies are generated. At this point, differentiated B cells leave the germinal center, becoming memory B cells and plasma cells. Plasma cells then leave the secondary lymphoid organ and migrate to the site of the tumour, surrounding it in the periphery of tertiary lymphoid structures, and meanwhile producing a large number of specific antibodies, exerting neutralization and ADCC effects.

B cells can differentiate into tumour-reactive plasma cells and produce specific antibodies during development (Fig. 1), which is determined by the diversity of B-cell receptors (BCR) [7]. The ability of different antigens to activate B cells is not the same and the ideal targets for various cancers are different. Thus, the selection of tumour antigens using objective criteria is key to determining the strength of the immunotherapy response.

The ideal antigen should be specifically expressed in cancer cells and not in normal cells and is necessary for the growth and survival of cancer cells with strong immunogenicity [8]. Tumour antigens mainly include tumour-associated antigens (TAAs) and tumour-specific antigens (TSAs). TAAs, including differentiation antigens, overexpression antigens, and cancer/testis antigens, are expressed in multiple cancers and in different cancer patients. Suitable TAAs should possess the following characteristics: differential expression between normal and tumour cells, involvement in the cell cycle, and correlation with cell survival [32]. TSAs are immunogenic peptides that originate from viral open reading frames (oncogenic viral antigens) or somatic mutations (neoantigens) rather than proteins expressed by the normal human genome [33, 34]. Table 1 introduces some typical TAAs and TSAs. Among TSAs, neoantigens, which are novel peptides, strongly activate immunity and have substantial clinical translational value [45]. Currently, the most studied shared mutations include KRAS, NRAS, BRAF, BCR-ABL translocation epitopes, tel-PDGFR β translocation epitopes, ALK, etc [46–50]. TSAs have been shown to possess high immunogenicity and can escape central thymic tolerance. Mutation-related antigens are ideal targets for immunotherapy [51]. Vinod *et al*. reported that personalised RNA vaccines based on neoantigens significantly improved the prognosis of pancreatic ductal adenocarcinoma [52]. This indicates the potential of B cells in neoantigen immunotherapy.

**Table 1.** Some typical tumour-associated antigens (TAAs) and tumour-specific antigens (TSAs)

Antigens	Gene	Clinical progress	Common cancer
TAAs	HER2(ERBB2)	III, IV	Breast cancer [35]
	EGFR	III	Non-small cell lung cancer, colorectal cancer, etc. [36]
	MAGE	I, II	Colon cancer, lung cancer, etc. [37]
	NY-ESO-1	I, II	Lung cancer, oesophageal cancer, liver cancer, ovarian cancer, etc. [38]
TSAs	BRAF V600E	II, III	Melanoma, colorectal cancer, etc. [39]
	EGFR T790M	II,III	Lung cancer, etc. [40]
	KRAS G12C	I, II	Lung cancer, colorectal cancer, etc. [41]
	HER2 (Y772_A775dup)	III	Breast cancer, stomach cancer, etc. [42]
	HPV E7	II, III	Cervical cancer, oral squamous carcinoma, etc. [43]
	EBV LMP1/LMP2	I	Nasopharyngeal carcinoma, etc. [44]

Tumour-reactive antibodies (TRAs) against MMP14 exist naturally in humans and have good immunotherapeutic effects against tumours [7]. Clinical results have also shown that plasma cells have a positive effect on prognosis [19]. Therefore, the use of tumour antigens to induce reactive plasma cells is of great help in immunotherapy. As previously mentioned, the use of neoantigens to activate and induce plasma cell differentiation is a promising strategy. Since neoantigens are only expressed by tumour cells, they prevent ‘off-target’ damage to normal tissue. Neoantigens are novel peptides derived from somatic mutations that theoretically prevent self-epitope tolerance [53].

## Significance of neoantigen-reactive plasma cells

In theory, new antigen-reactive plasma cells and antibodies have extremely low risks, except for antibodies that recognise self-antigens, and may cause autoimmune diseases. The interaction of neoantigen-reactive plasma cells with antibody therapies can enhance tumour-specific immune responses and improve the efficacy of and tolerance to antibody therapies. The synergistic effects and advantages of the two can provide a theoretical basis for individualised tumour treatments and the development of new immunotherapy strategies.

In addition, neoantigen-reactive B cells greatly contribute to T cells immune response. Cui *et al*. [20, 21] observed that tumour-specific B cells driven by neoantigen interact with CD4 T follicular helper cells and enhance the effector CD8 T cells function in lung adenocarcinoma (LUAD), which are necessary for tumour control [54].

As mentioned above, tumour-specific plasma cells are mainly derived from SHMs and exhibit a high degree of diversity [7]. Such plasma cells secrete IgG1, which functions as the primary tumour-killing antibody and promotes TLS formation [3].

Therefore, preparing neoantigen-reactive plasma cells and high-affinity tumour-specific antibodies and transfusing them into patients in large quantities can compensate for the shortcomings of humoral immunity in clinical practice and greatly improve treatment efficacy.

## Perspective of neoantigen-reactive plasma cell

Tumour-infiltrating lymphocytes (TILs) play crucial roles in anti-tumour processes. They can recognise and distinguish between normal and cancer cells, break through the cancer cell wall, and infiltrate cancer tissues, leading to the weakening and death of cancer cells. Based on the ability of TILs to recognise tumour-specific antigens, adoptive cell therapy (ACT) has emerged as a therapeutic approach that utilises the immune cells of patients to discover and eliminate tumour cells [55, 56].

### Idea of screening neoantigen-reactive plasma cell

**Figure 2.**
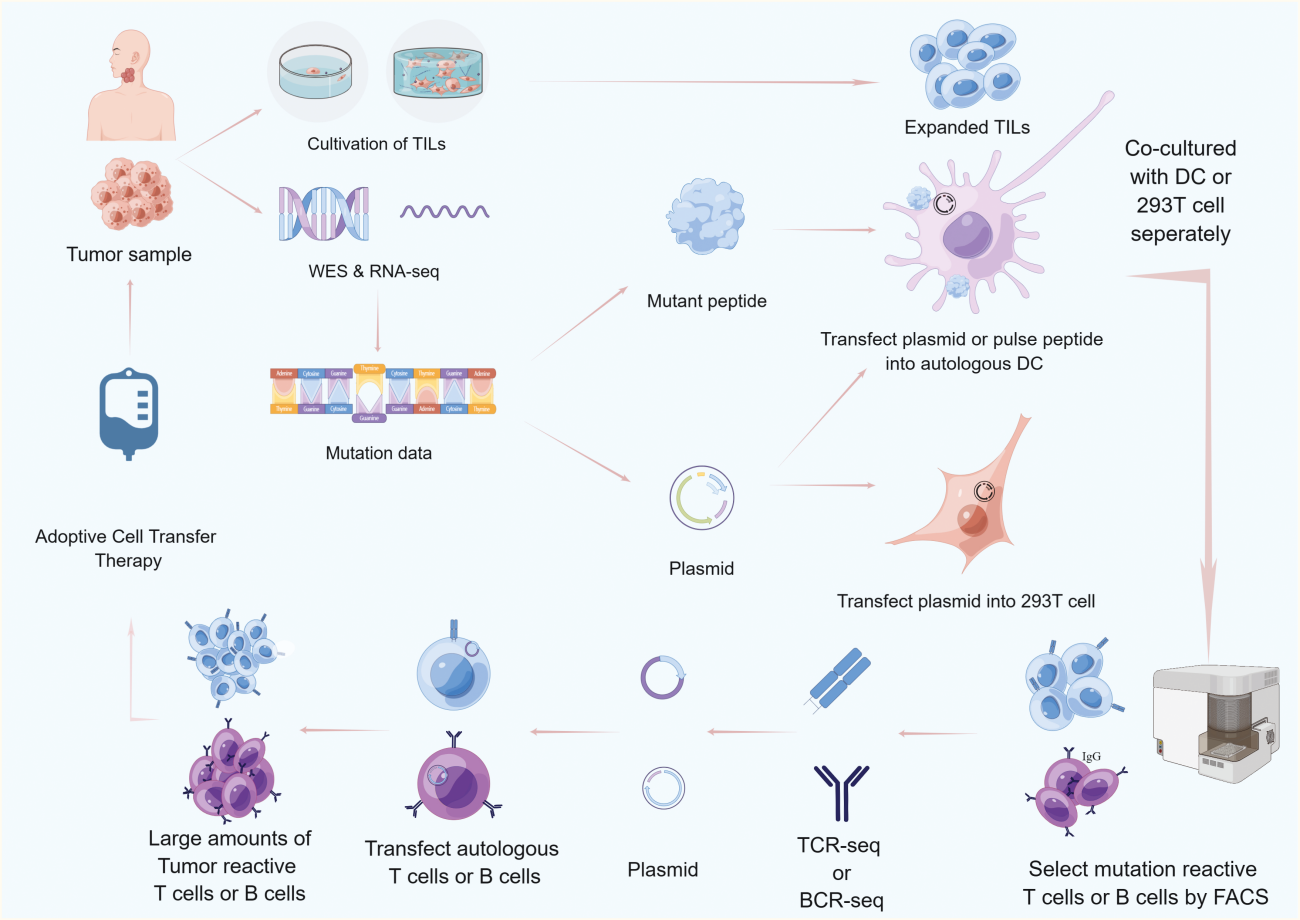
Screening of neoantigen-reactive TILs. Tumour samples are obtained from the patient, with a portion of the samples being separated and cultured to obtain amplified TILs. Another portion is used for WES and RNA-seq to acquire mutation information for synthesising mutated peptides and plasmids. The plasmids are transfected into dendritic cells, and the mutated peptides are pulsed onto dendritic cell surfaces, which are cocultured with TILs to simulate the MHCI and MHCII pathways of T-cell immune responses. Through transfecting plasmids into 293T cells, recombinant protein is expressed and cocultured with TILs to simulate B-cell immune responses. Activated T cells and B cells are sorted by flow cytometry for TCR-seq and BCR-seq to obtain sequence information and construct plasmids. The plasmids are transfected into autologous T cells or B cells, respectively to obtain tumour-reactive T cells or B cells containing specific TCR or BCR. These cells are expanded *in vitro* and adoptive transferred into patient for personalised treatment.

Highly specific plasma cells against neoantigens are undoubtedly effective tools for killing tumours. Currently, there are no relevant studies or reports on the screening of neoantigen-reactive plasma cells. However, a method called FucoID, developed in 2020, offers an effective approach for identifying tumour-targeting T-cells. This method involves loading fucosyltransferases onto dendritic cells, which when presenting tumour-specific material to desired T cells, transfers a label to the antitumour cells. Neoantigen-reactive T-cells can then be screened using fluorescent probes [57]. In 2022, Rosenberg improved the process for obtaining neoantigen-reactive T cells [58]. First, the mutation sites were obtained by sequencing the tumour samples. Plasmids expressing mutated peptides and synthetic long mutant peptides were transfected and pulsed into autologous dendritic cells. After co-culturing with tumour-infiltrating lymphocytes, the activated T cells were screened using flow cytometry. Specific TCR sequences were obtained using TCR sequencing. Secondly, tumour-infiltrating lymphocytes were sequenced and labelled with specific TCR sequences obtained earlier to confirm the consistency of antigen reactivity. Finally, autologous T cells were transfected with the screened TCR by reverse transcription and co-cultured with dendritic cells presenting the mutated epitopes. T-cell activation was evaluated by IFN-γ ELISPOT. These steps can generate a large number of neoantigen-reactive T-cells for adoptive transfer therapy to treat patients (Fig. 2).

There are many similarities between T and B cells in terms of antigen recognition, suggesting that B-cell immunotherapy strategies could be developed by referring to the pattern of T cells. Both of them have variable regions and constant regions, which determine their antigen specificity and species specificity. And the antigen-specific recognition region is CDR3, deriving from gene rearrangement. However, the difference in antigen recognition between T cells and B cells should also be fully considered. T cells epitopes are linear short peptides, while most of the B cells epitopes have spatial conformation, which adds difficulty to predict B cells associated neoantigens. At all events, advances in neoantigen-reactive T cells provide references for researching neoantigen-reactive plasma cells, including screening process for neoantigens, acquisition of tumour-reactive B cells, and construction of engineered B cells.

By collecting clinical tumour samples, whole-exome sequencing (WES) and annotation of tumour cells can be performed to identify non-synonymous mutation sites. Plasmids containing mutated peptide sequences were constructed. Using recombinant expression technology, neoantigens can be expressed and secreted into the extracellular environment and co-cultured with B cells to determine their activation status. Activated B cells can be sorted using flow cytometry, and BCR sequencing can be performed to obtain specific BCRs for comparison with the annotation results. The specific BCRs obtained can be connected to specific vectors for transfection into B cells and co-cultured with the corresponding antigen peptides to detect B cell activation. Ideally, successfully transfected B cells can be activated to obtain neoantigen-reactive B cells and prepare corresponding antibodies.

As earlier mentioned, newly identified antigen-reactive plasma cells and antibodies can address some of the limitations of immunotherapy. Simultaneously, personalised treatment has become a trend. Therefore, the research and preparation of tumour-specific antibody libraries hold great significance.

### Challenges and road ahead

The development of accurate epitope prediction algorithms and the optimisation of validation tools are key tasks for personalised cancer immunotherapy, especially when dealing with novel antigens. Currently, there is a notable gap in the development of MHC class II epitope prediction algorithms. Predicting the affinity of patient HLA alleles remains a major challenge and requires significant support in terms of personnel, resources, and funding.

Moreover, subsequent experimental validation is required to predict a large number of peptide epitopes. Efficient screening of antigenic epitopes from a large pool of candidate peptide sequences is a major technical challenge. There’s a well-known technology named serological analysis of recombinantly expressed cDNA clones (SEREX), which can identify tumour antigens at the molecular level. However, this technology cannot fold antigen properly and make construct post-transcriptional modifications, while three-dimensional structure is essential for the recognition of B cell epitopes. Therefore, SEREX alone may not be suitable for B cells antitumour immunity research, and extensive research is required to screen for antigens with strong immunogenicity and immune reactivity to validate the affinity and specificity of antibodies.

Even though, the first engineered B cell had been reported to be put into in Human Clinical Trial by Immusoft on 15 Dec 2023. Other biotechnology companies, like Be Biopharma and Walking Fish Therapeutics both contribute to engineered B cells in oncology and other diseases.

Overall, this area represents a new frontier in tumour immunotherapy. Despite the many challenges ahead, significant breakthroughs are expected in the near future.

## Data Availability

Not applicable.

## Abbreviations

ALK-1: activin receptor-like kinase-1
ADCC: antibody-dependent cellular cytotoxicity
ASCs: antibody-secreting cells
CICs: circulating immune complexes
LAs: lymphoid aggregates
ORR: objective response rate
SHM: somatic hypermutation
TAAs: tumour-associated antigens
TILs: tumour-infiltrating lymphocytes
TRAs: tumour-reactive antibodies
TRPCs: tumour-reactive plasma cells
TSA: tumour-specific antigen
WES: whole-exome sequencing.
